# Drought dominates the interannual variability in global terrestrial net primary production by controlling semi-arid ecosystems

**DOI:** 10.1038/srep24639

**Published:** 2016-04-19

**Authors:** Ling Huang, Bin He, Aifang Chen, Haiyan Wang, Junjie Liu, Aifeng Lű, Ziyue Chen

**Affiliations:** 1 State Key Laboratory of Earth Surface Processes and Resource Ecology, College of Global Change and Earth System Science, Beijing Normal University, Beijing 100875, China; 2 Department of Earth Sciences, University of Gothenburg, Gothenburg 450 30, Sweden; 3 College of Global Change and Earth System Science, Beijing Normal University, Beijing 100875, China; 4 Key Laboratory of Water Cycle and Related Land Surface Processes, Institute of Geographic Sciences and Natural Resources Research, CAS, Beijing 100101, China

## Abstract

Drought is a main driver of interannual variation in global terrestrial net primary production. However, how and to what extent drought impacts global NPP variability is unclear. Based on the multi-timescale drought index SPEI and a satellite-based annual global terrestrial NPP dataset, we observed a robust relationship between drought and NPP in both hemispheres. In the Northern Hemisphere, the annual NPP trend is driven by 19-month drought variation, whereas that in the Southern Hemisphere is driven by 16-month drought variation. Drought-dominated NPP, which mainly occurs in semi-arid ecosystems, explains 29% of the interannual variation in global NPP, despite its 16% contribution to total global NPP. More surprisingly, drought prone ecosystems in the Southern Hemisphere, which only account for 7% of the total global NPP, contribute to 33% of the interannual variation in global NPP. Our observations support the leading role of semi-arid ecosystems in interannual variability in global NPP and highlight the great impacts of long-term drought on the global carbon cycle.

Terrestrial ecosystems, which are a substantial sink for carbon and absorb approximately of one-quarter of annual anthropogenic emissions, dominate the interannual variability in atmospheric CO_2_ concentrations. Regional and global scale observations and modeling studies have revealed drought-induced decreases in plant growth and release of carbon by terrestrial ecosystems^1^,^2^,^3^. For example, drought was found to be the main reason for the decrease in global terrestrial net primary production (NPP) over the past decade^2^. This observation has raised great concern about the possibility of amplified climate warming due to impacts on the global carbon cycle^4^. However, previous studies have mainly focused on the effects of drought on specific ecosystems or the global ecosystem as a whole, and have not considered differences among ecosystems. Thus, the true contribution of drought to the variation in terrestrial net primary production is unclear. Observational and modeling studies have both suggested that drought will continue to increase in the future^4^,^5^. Hence, the monitoring and systematic analysis of the response of global NPP to drought is crucial to reduce uncertainty in projections of carbon cycling in terrestrial ecosystems.

Quantifying the influence of drought on the terrestrial carbon cycle requires an understanding of the physiological response of growth activity to drought stress. Resistance and resilience to water deficit and environmental conditions differ greatly among ecosystems, causing great divergences in the response of vegetative growth to drought^6^,^7^. According to a global examination of the response of vegetative growth to drought, humid biomes tend to respond to drought on relatively short timescales compared with semi-arid and sub-humid biomes^8^. In addition, the non-linear response of specific biomes to drought duration and intensity confounds the identification of the drought-induced turning point or the point at which an ecosystem is converted from a carbon sink to source^9^. Thus, considering multiscalar drought indices is important for investigating the relationship between drought and terrestrial NPP.

Here, we examine the relationship between drought and interannual variation (IAV) in global NPP considering variable timescales of drought. Our analyses are based on a multiscalar drought index, the Standardized Precipitation Evapotranspiration Index (SPEI)^10^, as well as global NPP data taken from the NASA Moderate Resolution Imaging Spectroradiometer (MODIS) MOD17 product and the AVHRR NDVI3g dataset. The SPEI is based on a climatic water balance and forced by climatic data. It accounts for the influences of precipitation deficit and atmospheric evaporative demand on drought severity^10^. Compared to the widely used Palmer Drought Severity Index (PDSI), SPEI can be used to identify water deficits and surplus conditions over variable time scales. The performance of SPEI was evaluated by comparing it with that of PDSI (Fig. S1).

## Results

### Relationship between drought and global NPP

**Figure 1:**
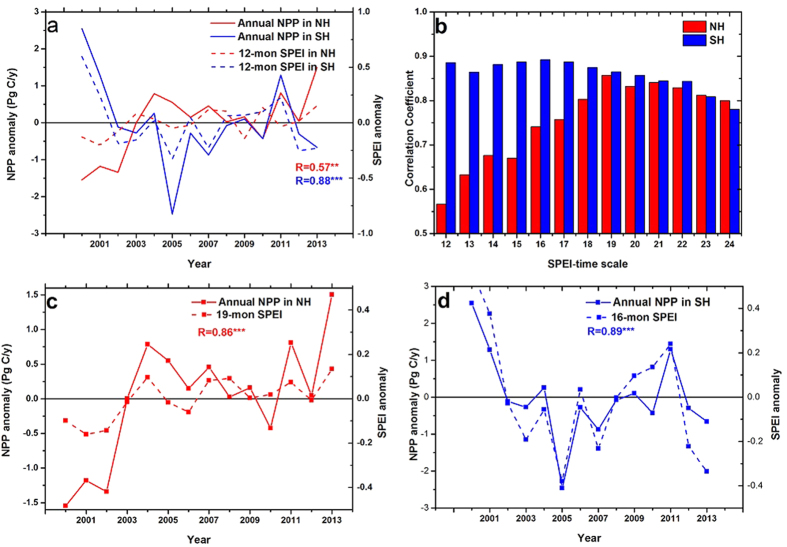
Interannual variations in anomalies of total NPP and average SPEI. (**a**) Relationships between annual NPP and annual SPEI in both hemispheres. (**b**) Correlation coefficients (Pearson coefficient, R) between annual NPP and 12- to 24-month SPEI. (**c**) Relationship between annual NPP and 19-month SPEI in the Northern Hemisphere. (**d**) Relationship between annual NPP and 16-month SPEI in the Southern Hemisphere. **Denotes 95% confidence level estimated with a *t*-test; ***denotes 99% confidence level estimated with a *t*-test.

Figure 1a shows the relationship between annual SPEI and NPP from 2000 to 2013. A significant positive relationship (R = 0.88, P < 0.01) between annual SPEI and NPP was identified in the Southern Hemisphere (SH), whereas the relationship was weaker (R = 0.57, P < 0.05) in the Northern Hemisphere (NH), indicating that NPP is more sensitive to drought in the SH than in the NH. This is consistent with the findings of an earlier study that used PSDI as an indicator of drought^2^. Considering the possible lag in the response of NPP to drought caused by physiological processes, the relationship between SPEI and NPP was re-examined by using SPEI over longer timescales. Interestingly, based on this multi-timescale analysis, drought was highly correlated with NPP in both hemispheres (Fig. 1b). In the NH, the relationship between SPEI and NPP increased sharply as the SPEI timescale was extended, reached the strongest correlation at 19 months (R = 0.86, P < 0.01), and decreased with further extension of the drought timescale (Fig. 1c). In the SH, the most robust positive relationship between SPEI and annual NPP was observed on a 16-month drought timescale (R = 0.89, p < 0.01), after which the correlation steadily decreased (Fig. 1d). To examine the potential impacts from autocorrelations in SPEI or NPP series on correlation coefficients between SPEI and NPP, the autocorrelations in relevant series had also been checked by using different numbers of lags, as presented in Table S1. No significant autocorrelation (p < 0.05) was observed for SPEI or NPP series, indicating the autocorrelation contribute little to P levels between SPEI and NPP. The above analysis indicates that 1) drought is a main driver of IAV in global NPP; and 2) long-term drought can better explain IAV in NPP than annual drought. Furthermore, annual NPP in the NH is related to cumulative water deficit over longer time scales than in the SH.

We selected 12 land-cover types according to the MODIS MCD12C1 land cover classification (Fig. S2 and Table S2) and examined the responses of NPP of specific ecosystems to multiscalar SPEI. We found large differences in the response of NPP to drought among ecosystems (Fig. S3). The strongest positive correlation between annual NPP and SPEI was observed in open shrubland in the SH (average correlation coefficient >0.6), followed by cropland (0.36), deciduous broadleaf forests (0.35), and grassland and savannas (0.34). Although croplands are human-dominated systems, the strong correlation suggests high sensitivity of agricultural vegetation to drought disturbances. This result could be supported by a recent finding that drought has great influences on global cereal production^11^. Positive but weak relationships were also found for deciduous needle leaf and mixed forests. The weakest and/or negative relationships were observed in evergreen broadleaf forests, woody savannas, and open shrubland in the NH, suggesting that these ecosystems did not respond strongly to changes in drought over long timescales. A common feature of these ecosystems is their location in very humid or high latitude regions of the NH, which supports the findings of Vicente-Serrano *et al.*^6^. For each biome, the maximum correlation coefficient occurred between NPP and 17-month SPEI, further illustrating that ecosystem NPP responds to drought over long timescales.

### Impact of drought on semi-arid ecosystem NPP

**Figure 2:**
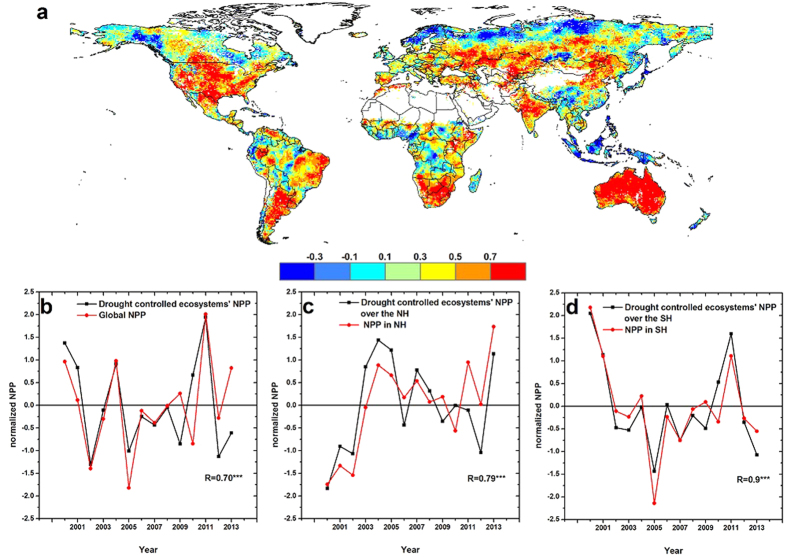
Role of drought-controlled ecosystems in interannual variation in global NPP. (**a**) Spatial pattern of maximum correlation coefficients (Pearson coefficient, R) between annual NPP and multi-timescale SPEI. The SPEI timescales range from 12 to 24 months. Red areas represent robust relationships at the 95% confidence level. (**b**) Variations in normalized NPP in drought-controlled ecosystems and global NPP. Drought-controlled ecosystems were defined as those ecosystems with a significant relationship between NPP and SPEI (P < 0.01). Variations in normalized NPP in drought-controlled ecosystems and NPP over (**c**) Northern Hemisphere and (**d**) Southern Hemisphere. ***Denotes 99% confidence level estimated with a *t*-test. This map was created using the ArcGIS 10.2 (http://www.esri.com/software/arcgis/arcgis-for-desktop).

The relationship between annual NPP and 12- to 24-month SPEI from 2000 to 2013 was examined for each grid cell, and the maximum correlation (Fig. 2a) and corresponding drought timescales were determined (Fig. S4). In the NH, strong positive correlations were identified in central Asia, the Indian Peninsula, and the southern part of North America. Significant positive relationships (P < 0.05) were identified in 51% of areas in the SH, mainly distributed in southern regions of South America and Africa, and Australia. Figure S4 shows the spatial patterns of SPEI timescales for which the maximum correlation between NPP and SPEI was found, indicating great differences in drought response among ecosystems.

In contrast with the global map of land cover classes (Fig. S2), we found that strong relationships between SPEI and NPP were mainly distributed in semi-arid ecosystems (Fig. 2a). Recent studies have suggested that IAV in the global carbon cycle is driven by semi-arid ecosystems^12^,^13^. This has raised speculation that the robust relationship between global SPEI and NPP is driven by semi-arid ecosystems. To examine this speculation, we extracted regional NPP in areas exhibiting significant SPEI–NPP relationships (P < 0.01) and compared the total regional NPP variability with the total global NPP (Fig. 2b). A strong correlation (R = 0.70, P < 0.01) was identified between annual variations in the NPP in drought-dominated ecosystems and global NPP, suggesting that drought drives global NPP through its influence on semi-arid ecosystems. This phenomenon was apparent in both hemispheres, but the relationship was stronger (R = 0.9, P < 0.01) in the SH than in the NH (R = 0.79, P < 0.01) (Fig. 2c,d). Further investigation revealed a weak relationship between variations in drought-controlled ecosystem NPP in the NH and global NPP (R = −0.06, P > 0.5), but a significant relationship between that in the SH and global NPP (R = 0.59, P < 0.05) (Fig. S5a,b). This indicates that the IAV in global NPP is mainly driven by semi-arid ecosystems in the SH, which is consistent with previous findings^2^,^12^,^13^. The autocorrelation coefficients of relevant series used in Figs 2b–d and S5 had also been calculated, and small coefficients (P > 0.05) demonstrate that autocorrelations in SPEI or NPP series had few influences on correlations between SPEI and NPP (Table S1).

### Contribution of drought-controlled ecosystem NPP to interannual variability in global NPP

**Figure 3:**
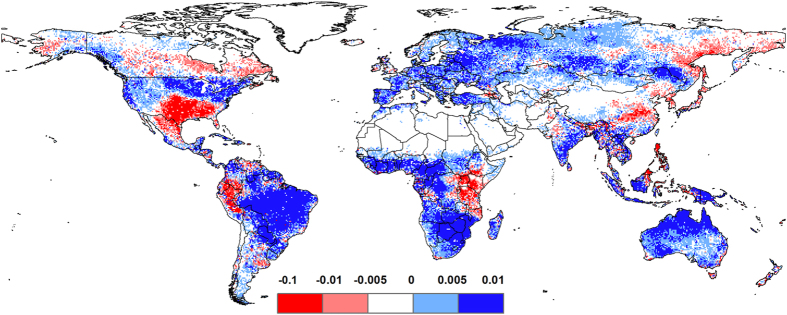
Local NPP contributions to global NPP interannual variation (%). Method for calculating contributions of regional IAV to global IAV was developed by Ahlström^12^. Blue and red areas indicate positive and negative contributions, respectively. This map was created using the ArcGIS 10.2 (http://www.esri.com/software/arcgis/arcgis-for-desktop).

To quantify the specific contribution of drought-controlled ecosystems to IAV, we employed the index proposed by Ahlström *et al.*^12^ to score the contribution of individual regions to IAV in global NPP. Drought-controlled ecosystems were determined as those exhibiting significant SPEI–NPP relationships (P < 0.01). These regions accounted for 29% of global NPP IAV during the study period, although their contribution to the total global NPP during this time (65.7 Pg C year^−1^) was only 16%. Moreover, drought-controlled ecosystems in the SH accounted for 33% of the global NPP IAV, whereas their total NPP only accounted for 7% of the total global NPP. These results provide further evidence that drought dominates the IAV in global NPP by controlling semi-arid ecosystems, especially in the SH (Fig. 3). Among the investigated biomes, evergreen broadleaf forests contributed to the largest proportion of global NPP IAV, followed by savannas.

To further examine the reliability of the relationship between satellite-observed NPP and SPEI presented above, we measured the correlation between NDVI (a proxy of vegetation productivity) and SPEI from 1982 to 2011. The two indices were significantly positively correlated (P < 0.05) over 85% of the Earth (Fig. S6), and the correlation coefficients had a similar spatial distribution to that of the annual NPP–SPEI correlation coefficients (Fig. 2a). In addition, the spatial distribution of the SPEI time intervals for which the maximum correlation between NDVI and SPEI was found (Fig. S7) was similar to that for which the maximum correlation between NPP and SPEI was found (Fig. S4), especially in Africa and Australia. Differences in SPEI timescales between Figs S4 and S7 had been calculated and shown in Fig. S8. One can find that the absolute differences were concentrated in the 0–3 month class over a majority of global vegetated areas. These findings suggest the following: 1) diverse responses of vegetation growth to drought under different climatic and environmental conditions; and 2) long-term drought can better explain vegetative growth than annual scale drought.

## Discussion

This analysis quantifies the contribution of drought to IAV in NPP and confirms the role of semi-arid ecosystems in the global terrestrial carbon cycle. A previous observation reported simultaneous annual drought and NPP trends, especially in SH ecosystems, whereas this study suggests that drought over longer timescales can better explain the variation in annual NPP. This can be explained by 1) the tolerance of vegetation in semi-arid ecosystem to short-term drought. According to a comprehensive investigation of xylem resistance to drought caused embolism, trees in semi-arid ecosystems were adapted to tolerate short-term water deficits due to the morphological or physiological acclimatization of species for coping with recurrent drought conditions^6^,^8^,^14^,^15^; 2) the lag in the effect of drought or related climatic variables on ecosystems, as supported by the recent finding that previous-year drought greatly contributes to changes in the current aboveground net primary production^16^; 3) the resistance and resilience of ecosystems to drought disturbances. Drought is mainly driven by precipitation and temperature. The legacy or lagged effect of these two variables and of drought on vegetative growth has been revealed in previous studies^7^,^9^,^17^,^18^. A recent investigation of forest recovery from severe drought based on *in-situ* data reported a 1–4 year legacy effect of drought on tree growth, and this effect was most prevalent in dry ecosystems^7^. Indirect impacts of drought on ecosystems (e.g., plant mortality^9^,^19^, wildfires^20^ and insect outbreaks^19^) can also cause a lag in the response of ecosystem NPP to drought; and 4) malfunction of physiological regulation mechanism of vegetation to cope with drought. The resistance and resilience of plants to drought through physiological activities such as promoting rainfall use efficiency (RUE)^21^, using deep roots to transport water, regulating hydraulic redistribution^22^ help plants survive short-term drought disturbances. However, such mechanisms malfunction when disturbances persist beyond certain thresholds and trigger growth declines or mortality^21^. For example, moderate drought stress can reduce photosynthetic rate and transpiration rate by restraining plant stomatal conductance, and then promote the RUE^23^,^24^. However, long-term drought should destroy the leaf photosynthesis system, causing sharp decrease in photosynthetic rate, decline in RUE, and even tree mortality due to hydraulic-failure or carbon starvation^25^.

This is likely another reason why global NPP is better correlated with long-term drought than annual drought. Although the above four potential mechanisms can explain the negative response of NPP to long-term drought, it is not yet known which one is dominant.

Another finding of this study is the great contribution of drought-controlled ecosystems to IAV in NPP, especially in SH ecosystems. In addition, a large proportion of the areas exhibiting a strong relationship between drought and NPP contained semi-arid ecosystems. This result is consistent with the previous finding of the major role of semi-arid ecosystems in IAV^12^,^13^. However, no clear relationship between NPP and drought was found in ecosystems located in cold northern areas or very humid ecosystems. This supports the previous finding of diverse responses of forest growth to drought in the NH. Forests in semi-arid areas tend to negatively respond to long-term drought, whereas those in cold areas and very humid ecosystems do not respond to long-term drought^6^. The reason for the weak response of humid ecosystems is that a positive water balance renders them unsusceptible to drought and/or enables them to rapidly recover from drought damage. The insensitivity of cold ecosystems to drought is likely because such areas are typically temperature-dominated^6^.

Knowledge from this study highlights the strong impact of drought on semi-arid ecosystems, the IAV in global NPP, and the global carbon cycle. Drought is projected to increase due to climate warming, which will likely exacerbate the degradation of fragile semi-arid ecosystems or covert semi-humid ecosystems into semi-arid ecosystems. Therefore, consistent monitoring and modeling studies of the dynamics of semi-arid ecosystems under climate change are of great importance for understanding the future global carbon cycle.

## Methods

### SPEI

We used the Standardized Precipitation Evapotranspiration Index (SPEI) developed by Vicente-Serrano *et al.*^10^ as an indicator of drought. This index is defined as a standardized variate of deviations in the current water balance with respect to the long-term balance. This newly defined drought index combines the sensitivity of PDSI to changes in evaporative demand and the flexible time-scale of SPI. The 1- to 24-month global SPEI dataset with a resolution of 0.5° from 1982 to 2013 was obtained from SPEIbase v.2.3^26^ (https://digital.csic.es/handle/10261/104742). In this database, potential evapotranspiration is calculated with the FAO-56 Penman-Monteith method. This dataset can reliably capture drought characteristics and identify drought impacts across a variety of ecosystems^10^,^27^. The SPEI dataset was compared with the global PDSI dataset (2.5° spatial resolution; 1982–2013) obtained from Dai^28^,^29^ (http://www.cgd.ucar.edu/cas/catalog/climind/pdsi.html) using a correlation analysis^22^,^23^. Except for a small proportion of intervals, significant positive relationships between annual SPEI and annual PDSI were found, suggesting high consistency between the two indices (Fig. S1).

### NPP and NDVI

The global annual MODIS NPP dataset (MOD17A3) (1-km spatial resolution; 2000–2013) was obtained from the Numerical Terradynamic Simulation Group (www.ntsg.umt.edu). Products of this dataset have recently been updated with a series of modifications^2^,^30^. This dataset has been widely used for the study of regional and global carbon cycles^21^,^31^,^32^, and is reliable with respect to magnitude and interannual variability^30^. The AVHRR NDVI3g dataset (0.083° spatial resolution; 15-day temporal resolution; 1982–2011) used here was developed by the Global Inventory Monitoring and Modeling Systems (GIMMS) project^33^. The annual NDVI series were generated using the maximum value composite (MVC) approach^34^. The NPP and NDVI datasets were aggregated to a 0.5° resolution to match the spatial resolution of the SPEI dataset.

### Global land cover data

Considering the great variability in the response of NPP to drought among ecosystems, we selected 12 types of biomes from the MODIS land cover product (MCD12C1) (0.05° spatial resolution). The biomes were classified according to the International Geosphere and Biosphere Programme (IGBP) land cover classification (Table S2). Open shrubland, which has a wide distribution from low latitudes to high latitudes, was divided into two categories with different climate-limiting conditions. Only areas (pixels) with a constant vegetation type during the period from 2001 to 2011 were used in this study. The global vegetation map is shown in Fig. S2.

### Statistical analysis

The impact of drought on terrestrial ecosystem productivity was investigated using a Pearson correlation analysis between global annual NPP and 12- to 24-month SPEI (2000–2013; 0.5° grid scales). A total of 23 coefficients were obtained for each time interval, and the maximum coefficient and corresponding drought timescale were determined. The interannual variation partitioning method^12^ was employed to quantify the contribution of drought to determine the IAV in global NPP. Using this method, IAV was partitioned into regions or grid cells, of which the relative contributions were quantified.

## Additional Information

**How to cite this article**: Huang, L. *et al.* Drought dominates the interannual variability in global terrestrial net primary production by controlling semi-arid ecosystems. *Sci. Rep.* **6**, 24639; doi: 10.1038/srep24639 (2016).

## Supplementary Material

Supplementary Information
