# Corrigendum: Drought dominates the interannual variability in global terrestrial net primary production by controlling semi-arid ecosystems

**DOI:** 10.1038/srep35126

**Published:** 2016-11-03

**Authors:** Ling Huang, Bin He, Aifang Chen, Haiyan Wang, Junjie Liu, Aifeng Lű, Ziyue Chen

Scientific Reports 6: Article number: 2463910.1038/srep24639; published online: 04 19 2016; updated: 11 03 2016

In this Article, Aifang Chen is incorrectly affiliated with ‘Department of Earth Sciences, University of Gothenburg, Gothenburg 450 30, Sweden’ The correct affiliation is listed below:

Department of Earth Sciences, University of Gothenburg, Gothenburg 405 30, Sweden.

