# Supplementary material for: Drought dominates the interannual variability in global terrestrial net primary production by controlling semi-arid ecosystems
Source: Sci Rep. 2016 Apr 19;6:24639. doi: 10.1038/srep24639 (PMC4835775; doi:10.1038/srep24639)

**Supplementary information**

**Drought dominates the interannual variability in global terrestrial net primary production by controlling semi-arid ecosystems**

Ling Huang^1^, Bin He^1*^, Aifang Chen^1^ , Haiyan Wang^1^, Junjie Liu^1^, Aifeng Lű ^2^ ,Ziyue Chen^1^

^1^College of Global Change and Earth System Science, Beijing Normal University, Beijing 100875,China.^2^Key Laboratory of Water Cycle and Related Land Surface Processes, Institute of Geographic Sciences and Natural Resources Research, CAS, Beijing 100101,China.

Correspondence and requests for materials should be addressed to B.H.([hebin@bnu.edu.cn](mailto:hebin@bnu.edu.cn))

**Table S1** Autocorrelation Coefficients of Studied Series

| Variables | Lag | | | |
| --- | --- | --- | --- | --- |
|  | 1 | 2 | 3 | 4 |
| SPEI in NH | 0.0604499 | -0.141788 | 0.113983 | -0.0023489 |
| SPEI in SH | 0.131216 | -0.118448 | -0.125719 | -0.168156 |
| NPP in NH | 0.375896 | 0.281969 | -0.210122 | -0.143088 |
| NPP in SH | 0.150574 | 0.0824501 | 0.0261637 | -0.202358 |
| drought-controlled ecosystems over global NPP | -0.0910511 | -0.460136 | 0.153279 | 0.0890157 |
| drought-controlled ecosystems over NH NPP | 0.222372 | 0.0395823 | -0.1956 | -0.261227 |
| drought-controlled ecosystems over SH NPP | 0.204694 | -0.166294 | -0.0971174 | -0.0895807 |
| Global NPP | -0.320436 | 0.0535959 | 0.119109 | 0.00577224 |
| NH NPP | 0.358523 | 0.2884 | -0.213526 | -0.13672 |
| SH NPP | 0.148396 | 0.0834598 | 0.0250191 | -0.204534 |

| **Table S2**  MODIS IGBP Land Cover Classification Types used in the study | |
| --- | --- |
|  | Land Cover Type |
| 1 | Evergreen Needleleaf forest |
| 2 | Evergreen Broadleaf forest |
| 3 | Deciduous Needleleaf forest |
| 4 | Deciduous Broadleaf forest |
| 5 | Mixed forest |
| N7 | Open shrublands(Northern Hemisphere) |
| S7 | Open shrublands(Southern Hemisphere) |
| 8 | Woody savannas |
| 9 | Savannas |
| 10 | Grasslands |
| 12 | Croplands |
| 14 | Cropland/Natural vegetation mosaic |


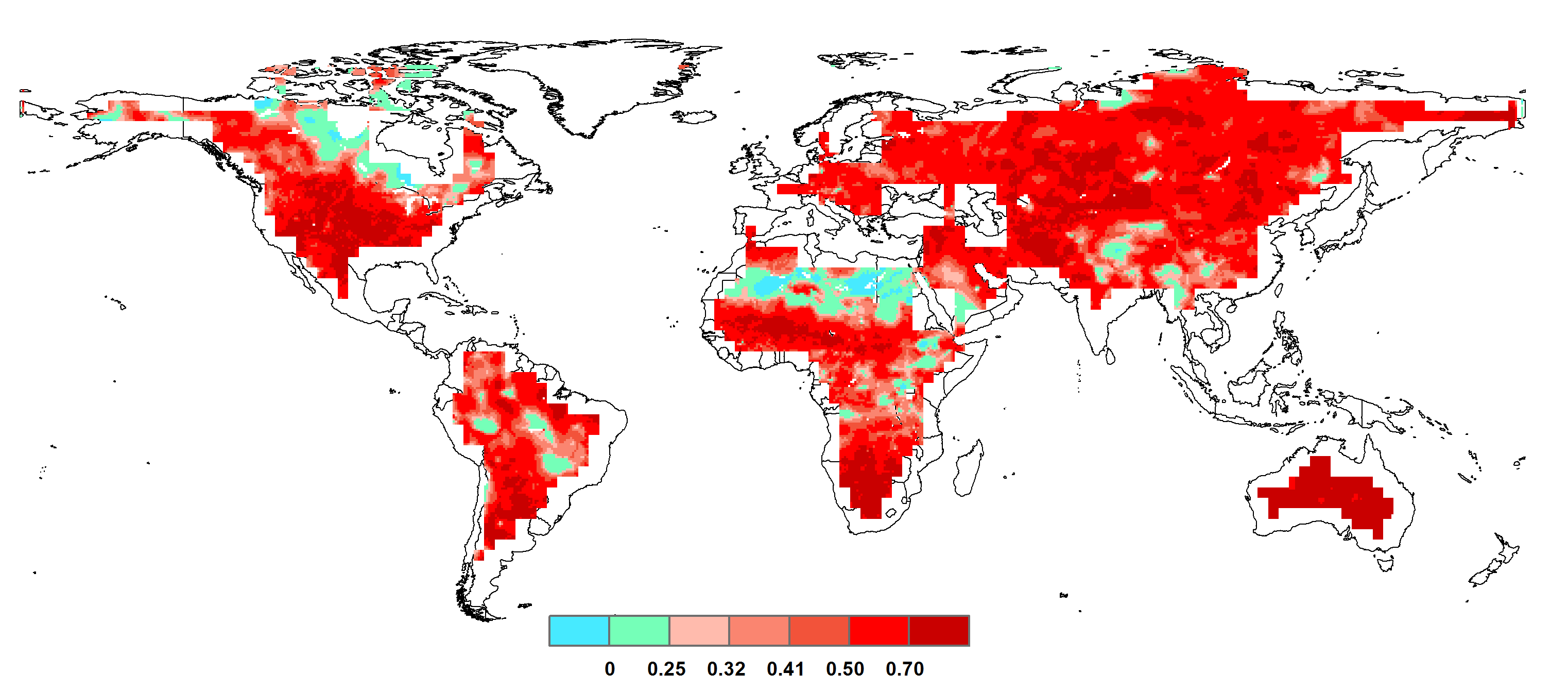


**Figure S1. Correlation coefficient (Pearson coefficient, R) between annual PDSI and 12-month SPEI for the period 1950-2012.** Correlation coefficients of 0.25, 0.32 and 0.41 indicate significant levels of 0.05, 0.01 and 0.001, respectively. PDSI data from Dai ^28, 29^. This map was created using the ArcGIS 10.2 (http://www.esri.com/software/arcgis/arcgis-for-desktop).
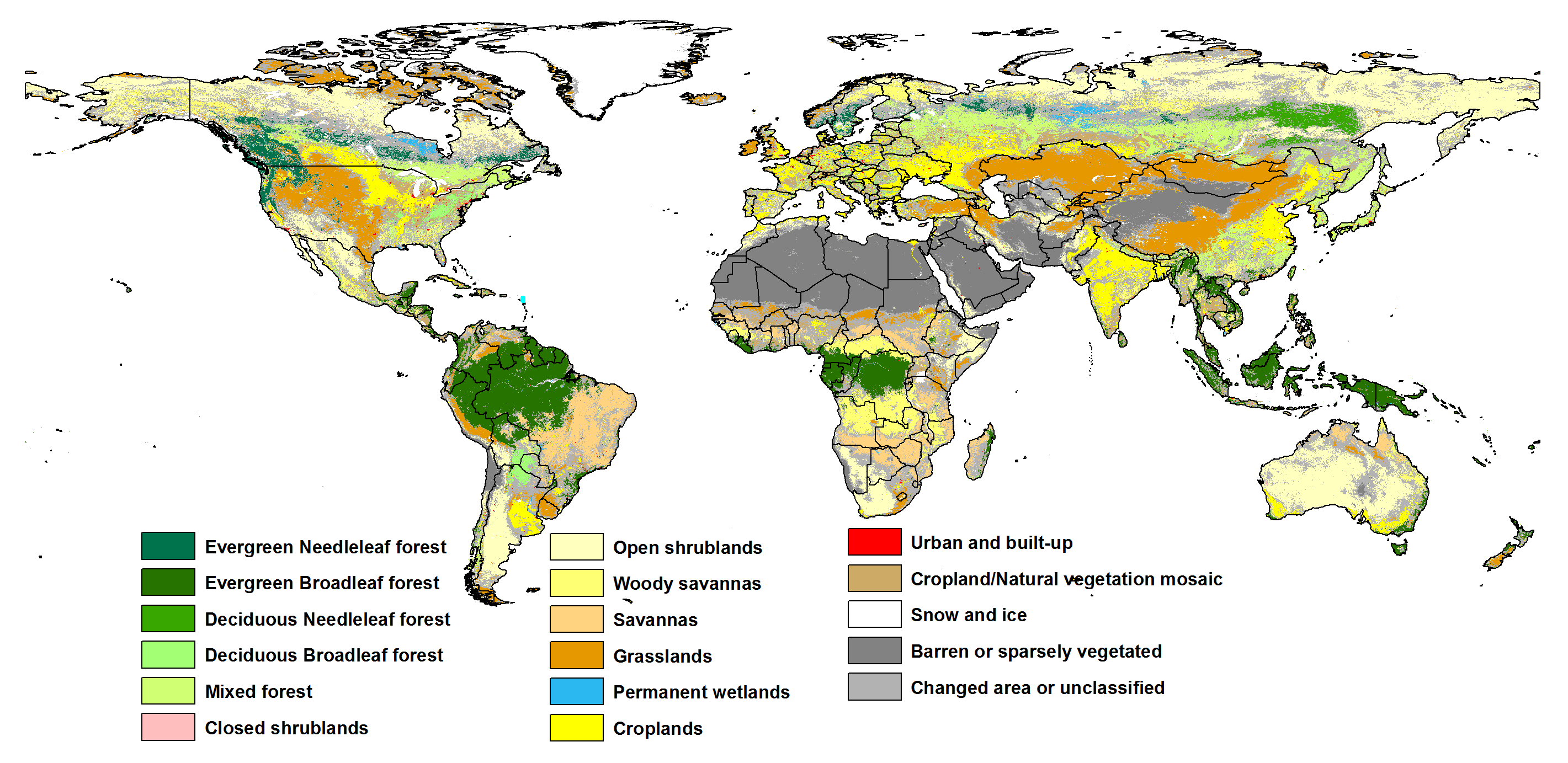


**Figure S2. Unchanged global land cover classification map.** This map was redrawn based on MODIS land cover product (MCD12C1) from 2001-2011 with IGBP (International Geosphere and Biosphere Programme) land cover classification. If pixels have changed vegetation type during the period 2001- 2011, corresponding areas were extracted and excluded for analysis. This map was created using the ArcGIS 10.2 (http://www.esri.com/software/arcgis/arcgis-for-desktop).


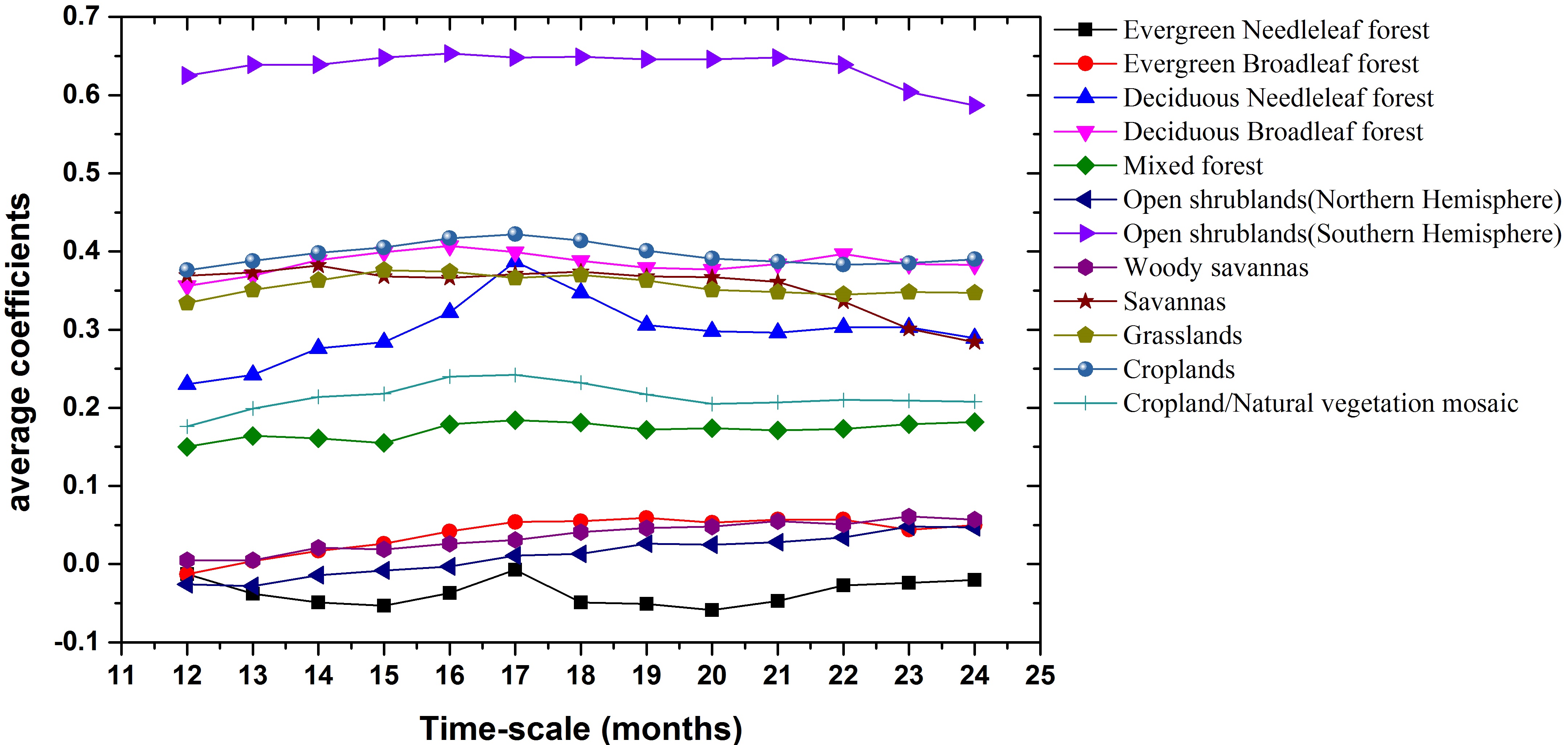


**Figure S3. Response of ecosystems to drought time-scales.** Average correlation coefficient

(Pearson coefficient, R) between annual NPP and muti-timescale SPEI for each 12 types of biomes. Time-scales of SPEI range from 12 to 24 months.


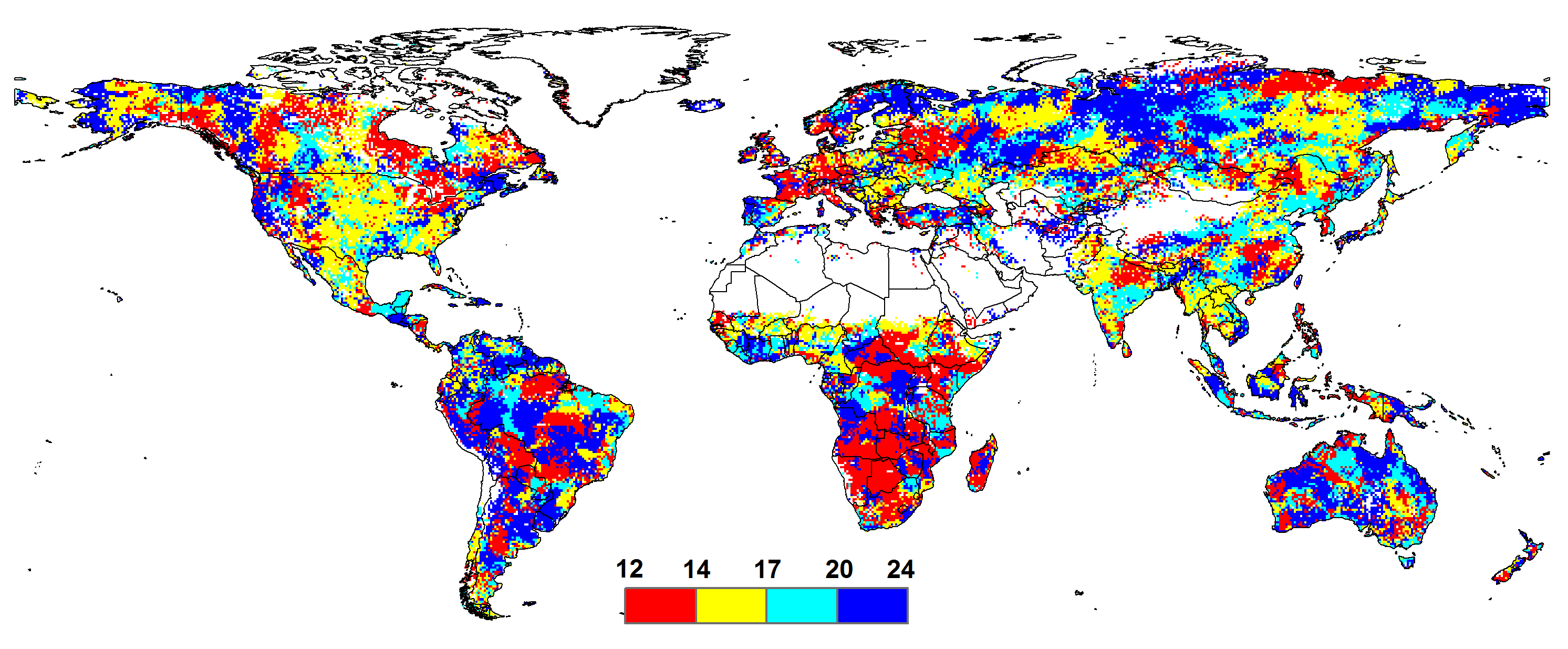


**Figure S4. Spatial patterns of SPEI time-scales at which the maximum correlation coefficient of NPP and SPEI was found.** The timescale of SPEI ranged from 12- to 24-months. This map was created using the ArcGIS 10.2 (http://www.esri.com/software/arcgis/arcgis-for-desktop).


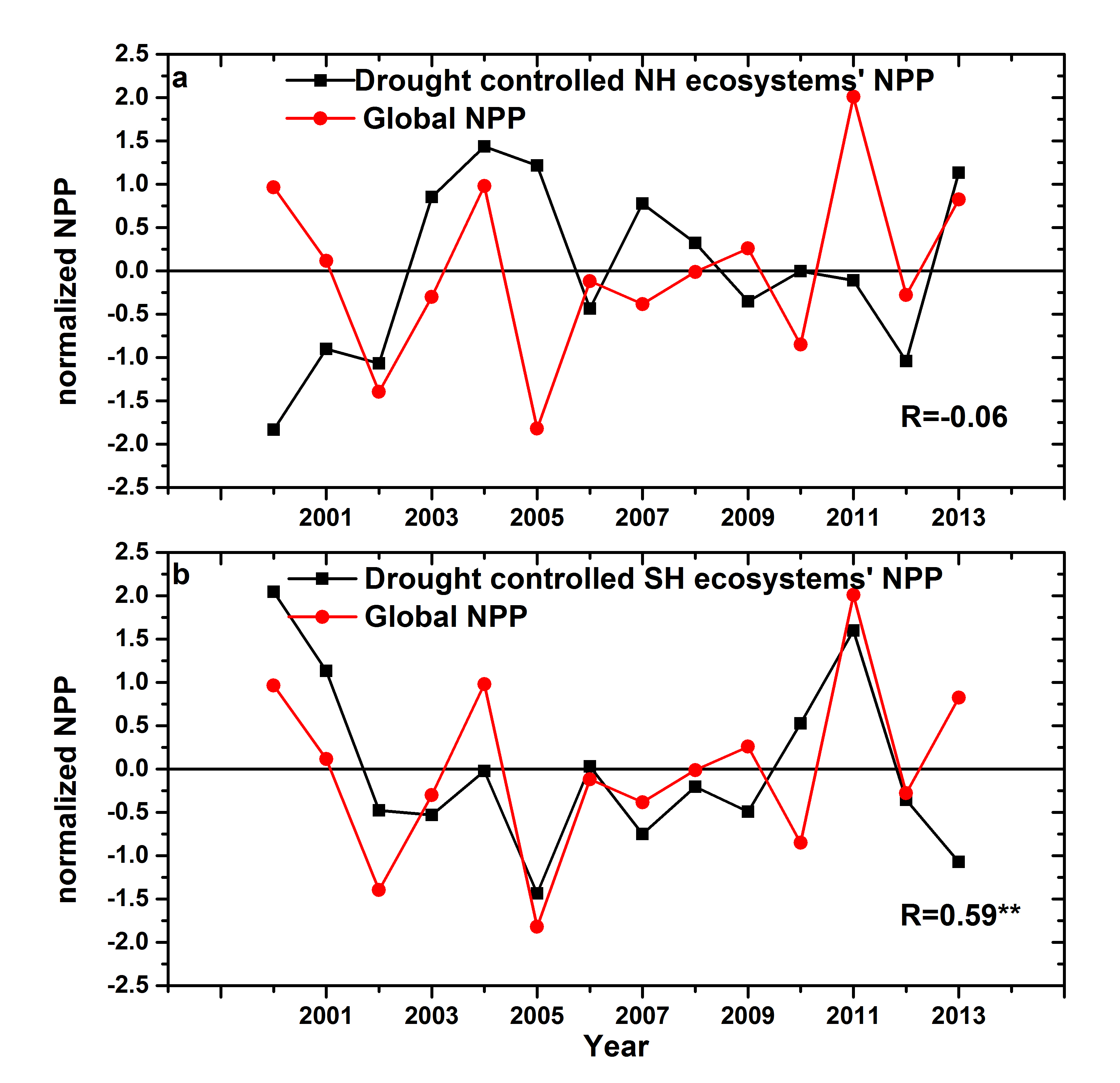


**Figure S5. Role of** **drought controlled ecosystems in interannual variation of global NPP.**

(a) Variations of normalized NPP in drought-controlled ecosystems over NH and global NPP.

(b) Variations of normalized NPP in drought-controlled ecosystems over SH and global NPP. Drought-controlled ecosystems were defined as those ecosystems with significant relationship between NPP and SPEI (P < 0.01). ** denotes 95% conﬁdence level estimated by a *t*- test.


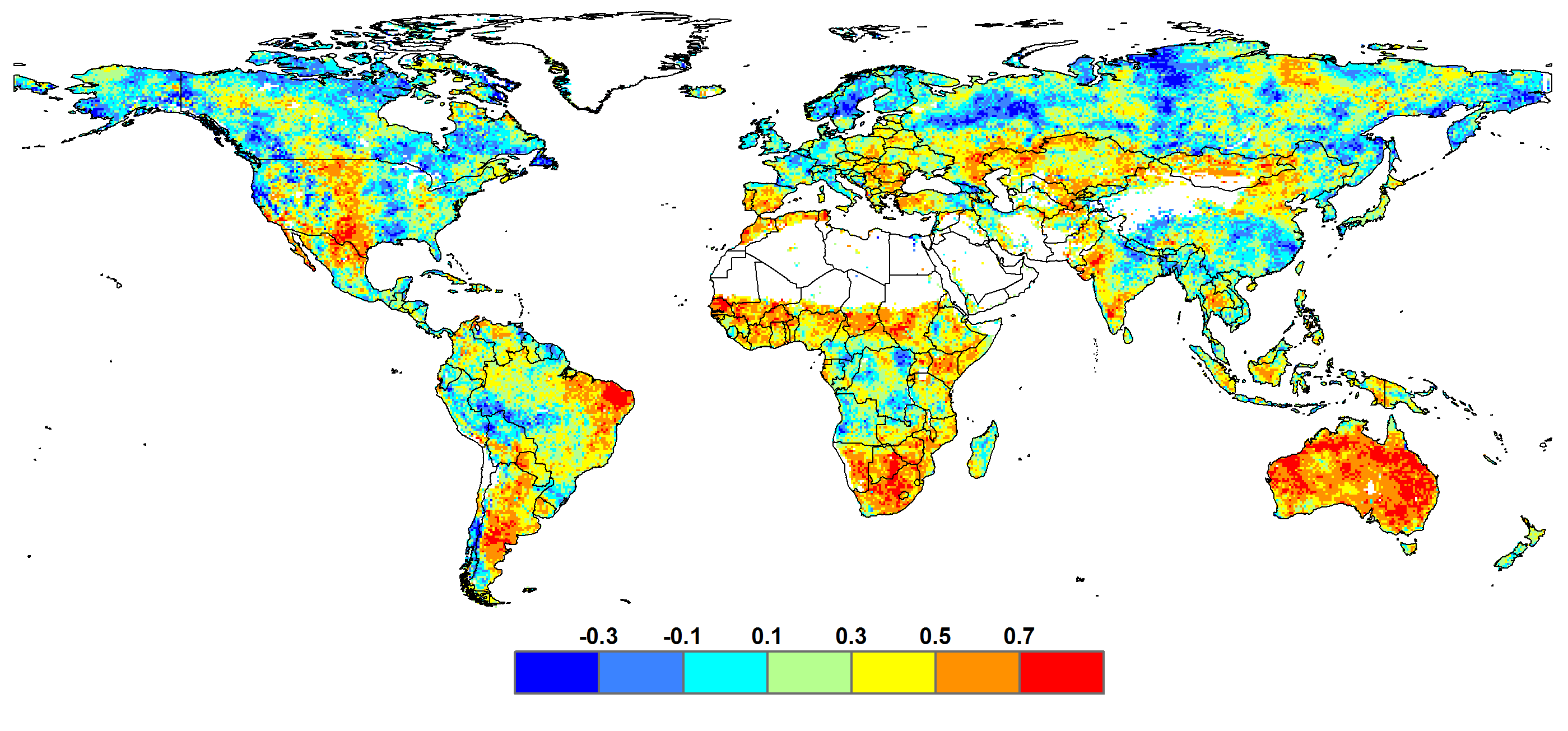


**Figure S6. Spatial pattern of maximum correlation coefficients between average annual NDVI and muti-timescale SPEI.** The timescales of SPEI range from 12- to 24-months. Red color represents robust relationships at 99% conﬁdence level. This map was created using the ArcGIS 10.2 (http://www.esri.com/software/arcgis/arcgis-for-desktop).


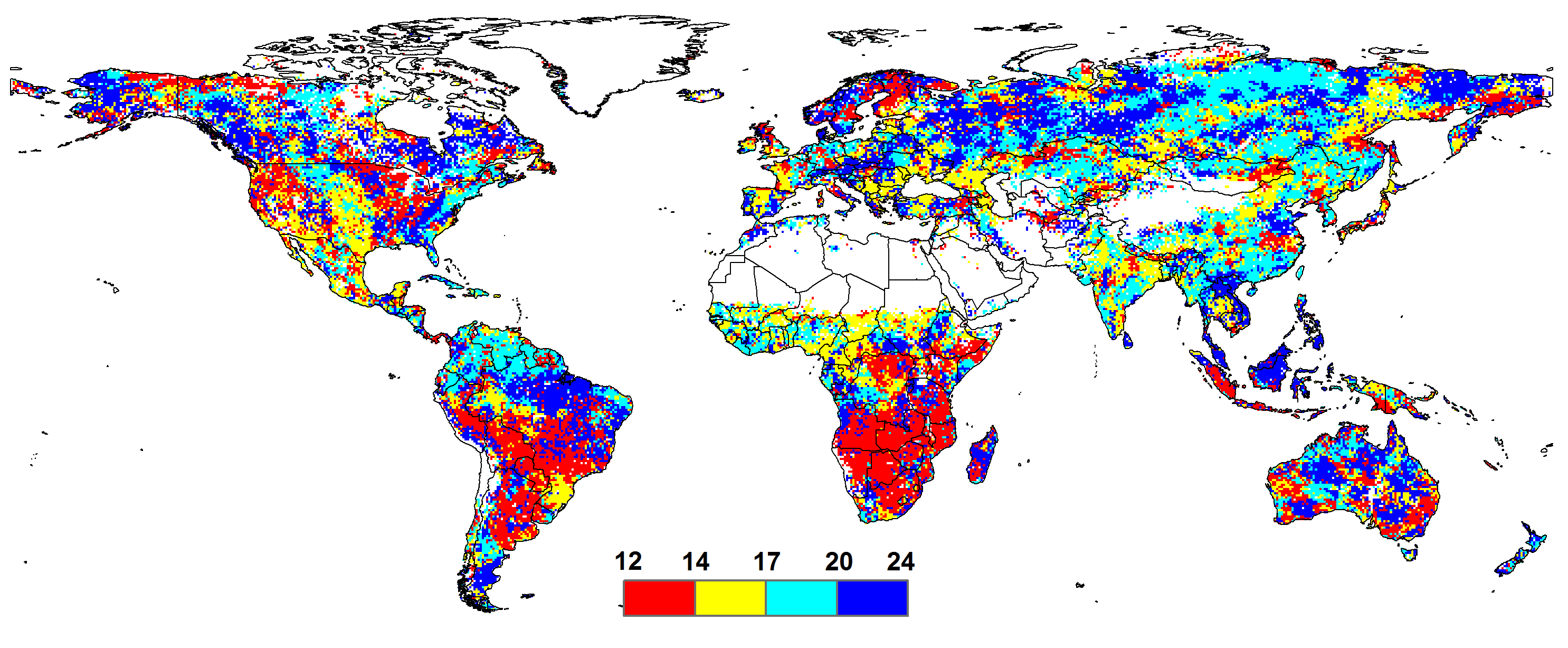


**Figure S7. Spatial patterns of SPEI time-scales at which the maximum correlation of NDVI and SPEI was found.** The timescale of SPEI ranged from 12- to 24-months. Desert and ice areas are not analyzed in this study. This map was created using the ArcGIS 10.2 (http://www.esri.com/software/arcgis/arcgis-for-desktop).

**Figure S8. Differences in SPEI timescales between Figure S4 and Figure S7.** The differences are expressed by absolute values. Desert and ice areas are not analyzed in this study. This map was created using the ArcGIS 10.2 (http://www.esri.com/software/arcgis/arcgis-for-desktop).


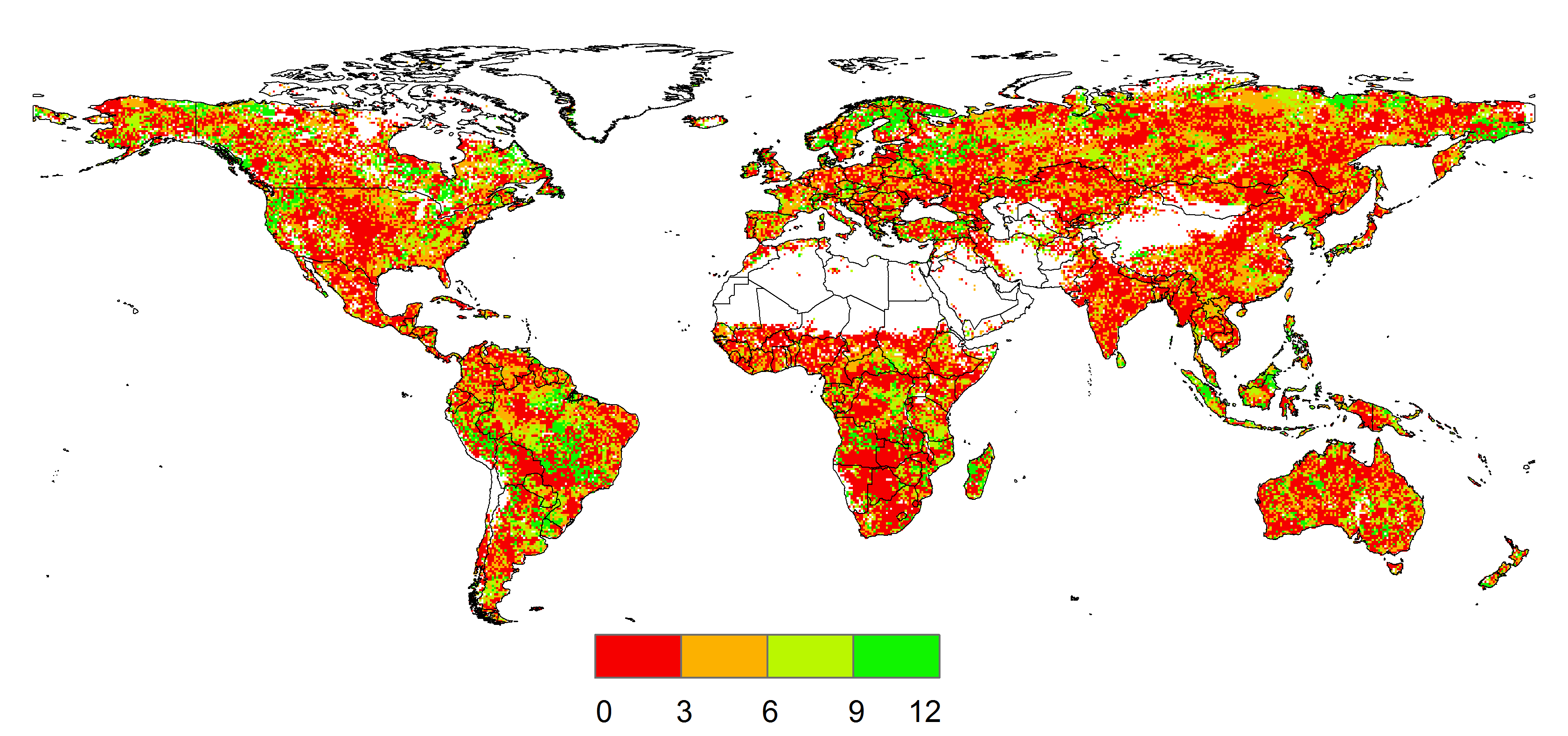

Supplement: Supplementary Information [file srep24639-s1.doc]
